# Functionalization of Molybdenum Disulfide via Plasma Treatment and 3-Mercaptopropionic Acid for Gas Sensors

**DOI:** 10.3390/nano10091860

**Published:** 2020-09-17

**Authors:** Won Seok Seo, Dae Ki Kim, Ji-Hoon Han, Kang-Bak Park, Su Chak Ryu, Nam Ki Min, Joon Hyub Kim

**Affiliations:** 1 Department of Control and Instrumentation Engineering, Korea University, 2511 Sejong-ro, Sejong-si 30019, Korea; wonseok0630@korea.ac.kr (W.S.S.); keypoint0460@korea.ac.kr (D.K.K.); kbpark@korea.ac.kr (K.-B.P.); 2 Department of Nanomechatronics Engineering, Pusan University, Busan, 2 Busandaehak-ro 63 beon-gil, Geumjeong-gu, Busan 46241, Korea; dafory@korea.ac.kr (J.-H.H.); scryu@pusan.ac.kr (S.C.R.)

**Keywords:** functionalization of 2D materials, molybdenum disulfide, gas sensors

## Abstract

Monolayer and multilayer molybdenum disulfide (MoS_2_) materials are semiconductors with direct/indirect bandgaps of 1.2–1.8 eV and are attractive due to their changes in response to electrical, physicochemical, biological, and mechanical factors. Since the desired electrical properties of MoS_2_ are known, research on its electrical properties has increased, with focus on the deposition and growth of large-area MoS_2_ and its functionalization. While research on the large-scale production of MoS_2_ is actively underway, there is a lack of studies on functionalization approaches, which are essential since functional groups can help to dissolve particles or provide adequate reactivity. Strategies for producing films of functionalized MoS_2_ are rare, and what methods do exist are either complex or inefficient. This work introduces an efficient way to functionalize MoS_2_. Functional groups are formed on the surface by exposing MoS_2_ with surface sulfur vacancies generated by plasma treatment to 3-mercaptopropionic acid. This technique can create 1.8 times as many carboxyl groups on the MoS_2_ surface compared with previously reported strategies. The MoS_2_-based gas sensor fabricated using the proposed method shows a 2.6 times higher sensitivity and much lower detection limit than the untreated device.

## 1. Introduction

As a 2D material, graphene has been demonstrated to exhibit differing electrical and optical properties depending on factors such as electrical and magnetic fields [1], strain [2], stacking geometry [3], and edge chirality [4,5] However, many have realized the applicability limitations of graphene since it does not have a bandgap, which has raised interest in transition-metal dichalcogenide (TMD) materials. TMD materials consist of hexagonal metal atoms (M) sandwiched between two chalcogen atomic layers (X), which form 3D crystals through the lamination of adjacent sheets through van der Waals interactions. TMD materials are potentially useful in electronics owing to their superconductivity [6] and charge density wave effects [7].

Monolayer and multilayer molybdenum disulfide (MoS_2_), one of the most studied TMDs, are semiconductors with direct/indirect bandgaps of 1.2–1.8 eV [8,9] and are highly attractive due to the considerable environment-dependent changes in their electrical [10,11,12,13], physicochemical [14], biological [15], optical [16], and mechanical properties [17]. Particularly, a field-effect transistor with monolayer MoS_2_ exhibited highly favorable electrical properties such as a mobility of 200 cm^2^/(V s), on/off ratio of 108, and sub-threshold swing of 70 mV/dec [11]. Since these electrical properties of MoS_2_ were revealed, related studies have increased, and research toward extending its applicability has focused on large-area deposition and growth [18,19,20,21] and functionalization [22,23,24,25]. While significant progress has been made toward large-scale MoS_2_, studies on functionalization techniques have yet to be actively conducted. Functional surface ligands consist of an anchor group that can attach to gas molecules, nanocrystals, biomaterials, etc. Particularly, functional groups can help dissolve the particles and provide adequate reactivity. Therefore, functionalization strategies for these materials are necessary. Detailed strategies must be established since some functionalization techniques for carbon nanotubes are not suitable for TMD materials.

Attempts have been intermittently made to introduce new MoS_2_ functionalization strategies. Recently, a report described an approach to directly and simply functionalize MoS_2_ using nitrilotriacetic acid [22]. However, the method requires a transition-metal cation with a high sulfur affinity and octahedral coordination since the direct anchoring of organic ligands to the sulfur surface is impossible. Although their new method was simple, the application of transition-metal cations complicates the process. Chen et al. and Chu et al. introduced methods for functionalizing MoS_2_ using 4-nitrobenzenediazonium tetrafluoroborate [23] and cysteine [24], respectively, after generating sulfur vacancies, but neither described how to generate the sulfur vacancies or gave detailed experimental methods. The direct synthesis of various organic functional groups on MoS_2_ thin films has also been reported [25], but the method is less efficient than the proposed method and lacks optimization.

## 2. Materials and Methods

Tri-layer MoS_2_ films grown through CVD were obtained from 6carbon (Shenzhen, China). Gold paste (C2041206P2) as a gas sensor pad was purchased from Gwent (Pontypool, UK). 3-Mercaptopropionic acid (MPA) was purchased from Sigma-Aldrich (St. Louis, MO, USA), and a 40 mM MPA solution was prepared using a mixture of 99% ethanol and deionized (DI) water.

The fabricated MoS_2_-based gas sensor was characterized by scanning electron microscopy (SEM) (Hitachi, Tokyo, Japan), and the change in resistance in response to the constituent gas concentration was measured using an LCR meter (Hioki, Nahano, Japan). The chemical composition for each experimental step was investigated using X-ray photoelectron spectroscopy (XPS) (Thermo, MA, U.S.A). The sulfur vacancies and MoS_2_ thickness after plasma treatment were measured by Raman spectroscopy (LabRAM, Horiba KOREA, Bucheon, Korea) and atomic force microscopy (AFM) (Shimadzu, Kyoto, Japan), respectively.

**Figure 1:**
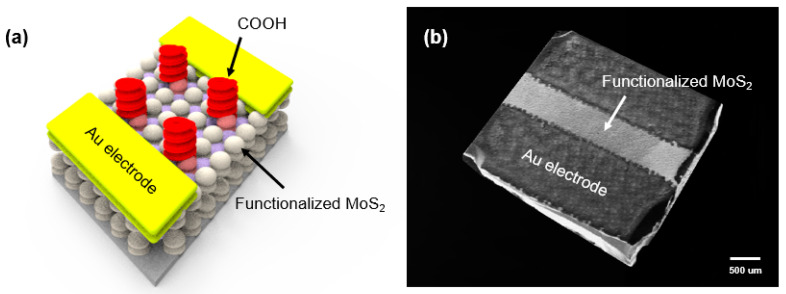
(**a**) Schematic representation and (**b**) SEM image of the gas sensor prepared with an MoS_2_ film functionalized by plasma treatment for 2 s at 10 W and exposure to an 3-Mercaptopropionic acid (MPA) solution overnight.

The MoS_2_ film grown by CVD on a 3 mm × 3 mm SiO_2_/Si substrate was plasma-treated at 10 W for 2 s in an Ar^+^ atmosphere to create sulfur vacancies. The plasma-treated MoS_2_ film was then exposed to the MPA solution overnight to form coordinate bonds between the HS groups in MPA and sulfur vacancies. To fabricate the gas sensor pad, the gold paste was screen-printed onto both ends of the functionalized MoS_2_ film. Figure 1 shows a schematic diagram and SEM image of the functionalized MoS_2_-based gas sensor.

## 3. Results and Discussion

**Figure 2:**
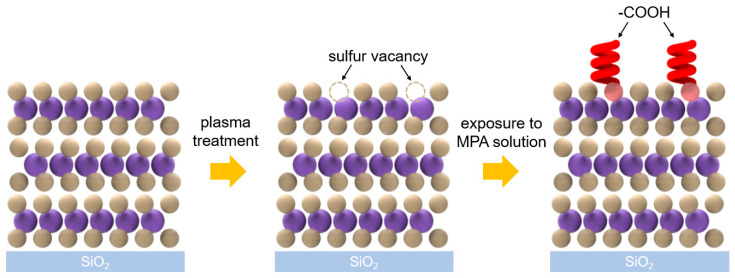
Schematic representation of the efficient and stable functionalization of an MoS_2_ film.

**Figure 3:**
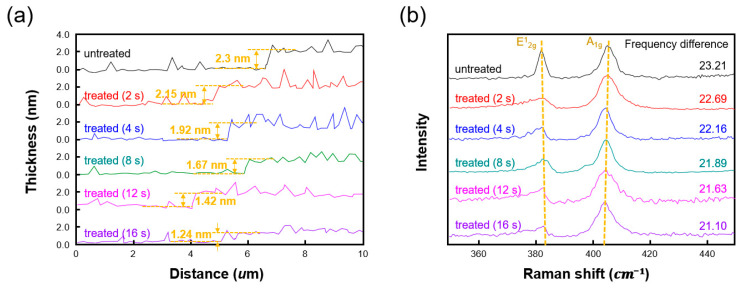
(**a**) Thickness and (**b**) Raman spectra of MoS_2_ films that were plasma treated for different durations. The frequency difference between Raman shifts indicates that the etching of the MoS_2_ film depended on the treatment time. In addition, the AFM results indicate that the sulfur vacancies were generated in 2 s.

The presence of functional groups on a sensing material is a crucial factor in determining its performance since they can increase the sensitivity. Figure 2 shows the functionalization process of the MoS_2_ films. The first step involves the generation of sulfur vacancies by plasma treatment. Since MoS_2_ is a 2D material, it is highly susceptible to physical damage, and thus a weak and chemically inactive plasma treatment should be performed. Since the minimum power of our home-built plasma equipment is 10 W, the plasma power was fixed at 10 W, and Ar ions were used as plasmons for their chemical inactivity. As shown in Figure 3a, AFM analysis was conducted to examine the physical changes in the MoS_2_ film over the treatment period. The thickness of a three-layer MoS_2_ film is theoretically 2.3 nm [8,9,26], which was the same as the thickness measured by AFM analysis. As the treatment time increased, the MoS_2_ film was physically etched at a rate of 0.6 Å/s. The etch rate of the Ar plasma was notably slower than those reported for previous studies using oxygen plasma [11], indicating that Ar plasma is more suitable for the generation of sulfur vacancies in MoS_2_ films.

Figure 3b shows the Raman spectra of the MoS_2_ films that were plasma treated for different durations. Numerous theoretical studies have indicated that the frequency difference between the E^1^_2g_ and the A_1g_ Raman modes is dependent on the thickness of the MoS_2_ film [18]. The layers of MoS_2_ are held together via van der Waals forces. As the number of layers decreases, less incident energy is lost through the vertical vibrations of the molecules, resulting in a decrease in the frequency of the A_1g_ peak [18,19,20,21]. Conversely, the E^1^_2g_ peak is affected more by the interatomic coulomb potential, which decreases with fewer layers due to the reduction in interactions between atoms. As a result, the E^1^_2g_ frequency increases. This increase in E^1^_2g_ frequency corresponding to horizontal vibrations is less sensitive to the number of layers than the decrease in A_1g_ frequency corresponding to vertical vibrations. The A_1g_ peak occurring above the bi-layer rather than a single layer is less sensitive to the MoS_2_ thickness. However, the E^1^_2g_ peak is sensitive to the MoS_2_ thickness. The reduced sensitivity to thickness of the E^1^_2g_ mode has been explained by the less-efficient inter-layer coupling of the in-plane phonons [27]. As the MoS_2_ becomes the generation of sulfur vacancies was confirmed to be 2 s, since the physical thickness of sulfur is 0.176 nm thinner, the E^1^_2g_ frequency increases and the A_1g_ frequency decreases, thereby reducing the frequency difference. This frequency difference for the MoS_2_ film treated for 8 s was reduced from 23.21 for the untreated film to 21.89 cm^−1^. A previous paper reported this frequency difference for a bilayer MoS_2_ film as 21.61 cm^−1^ [28]. Therefore, the Raman analysis indicates that the treatment time required to etch one layer was 8 s in the proposed method. Thus, to form sulfur vacancies in a 3-layer MoS_2_ film without removing a layer, a treatment time of less than 8 s is required. As demonstrated by the AFM analysis (Figure 3a), the treatment time for the generation of sulfur vacancies was confirmed to be 2 s, since the physical thickness of sulfur is 0.176 nm.

**Figure 4:**
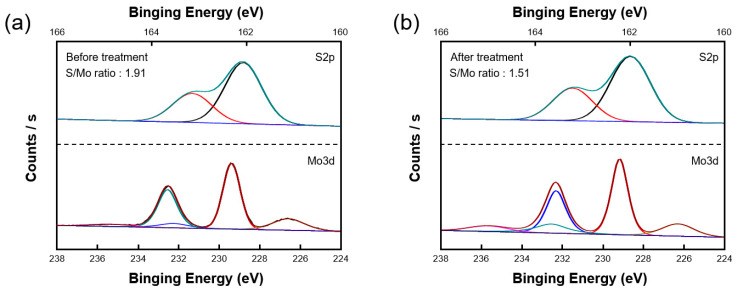
Chemical compositions of MoS_2_ films (**a**) before and (**b**) after plasma treatment. After the plasma treatment, the S/Mo ratio decreased from 1.91 to 1.51, indicating the creation of sulfur vacancies.

The XPS S 2p and Mo 3d deconvolutions further supported the generation of sulfur vacancies by the plasma treatment (Figure 4). The Mo 3d52 and S 2p32 peaks of the MoS_2_ film appeared at 229.3 and 162.2 eV, respectively, which have previously indicated an MoS_2_ film with three layers [25]. The S/Mo ratio can be calculated as the area ratio of the S 2p orbital representing the number of S atoms and the Mo 3d orbital representing the number of Mo atoms. Using the integral, the area of each orbital according to the XPS result was calculated, and the S/Mo ratio was calculated to compare before and after plasma treatment. The untreated MoS_2_ film showed the ideal S/Mo ratio of 1.91, while that of the plasma-treated MoS_2_ film was 1.51, demonstrating the formation of sulfur vacancies [29].

**Figure 5:**
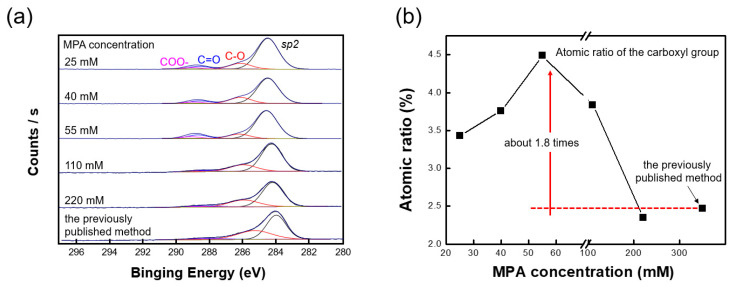
(**a**) C 1s deconvolution curves and (**b**) atomic ratios of films treated with different MPA concentrations. The MPA concentration was optimized at 55 mM. The MoS_2_ film treated at the optimized concentration had 1.8 times more carboxyl groups than the film prepared by the previously reported strategy.

For the functionalization of the MoS_2_ surface, the MoS_2_ film with sulfur vacancies was exposed to an MPA solution. MoS_2_ has been used as a hydrodesulphurization catalyst because surface sulfur vacancies tend to form covalent bonds with sulfur-containing groups. As a result, molecules with thiol functional groups chemically bond to the sulfur vacancies formed on the MoS_2_ surface [30,31]. The chemical components of the MoS_2_ films with sulfur vacancies treated in solutions of MPA at different concentrations were measured by XPS to determine the optimized MPA concentration (Figure 5a). The measured absorbance spectra presented four peaks; the first at 285 eV was assigned to sp^2^-hybridized carbon atoms, and the other three at 286.06, 288.51, and 288.97 eV were assigned to oxygen-containing hydroxyl (C−O), carbonyl (C=O), and carboxyl groups (−COO), respectively. The presence of these oxygen-containing group features implies that the functional groups were stably bonded to the MoS_2_ film [32]. From the XPS results for different MPA concentrations, the atomic ratio corresponding to carboxyl groups increased for concentrations up to 55 mM and then decreased, potentially because high concentrations of MPA may form inter-MPA disulfide bridges (Figure 5b).

Figure 5 also compares the effectiveness of the proposed and previous method for the formation of functional groups. The exfoliation process by *n*-butyllithium deforms the crystal structure of MoS_2_ and introduces defects mainly at the edges of the exfoliated 2D material [25]. Conversely, the defects introduced by the plasma treatment were evenly generated on the MoS_2_ surface, and thus more defects were created compared with the exfoliation process. As previously mentioned, since thiol functional groups covalently bond to the defect sites, the number of defect sites is a crucial factor in the functionalization of the nanosheets. As illustrated by the C 1s deconvolution in Figure 5, approximately 1.8 times more carboxyl groups were produced using the proposed strategy compared with the amount generated by the previously published methods.

**Figure 6:**
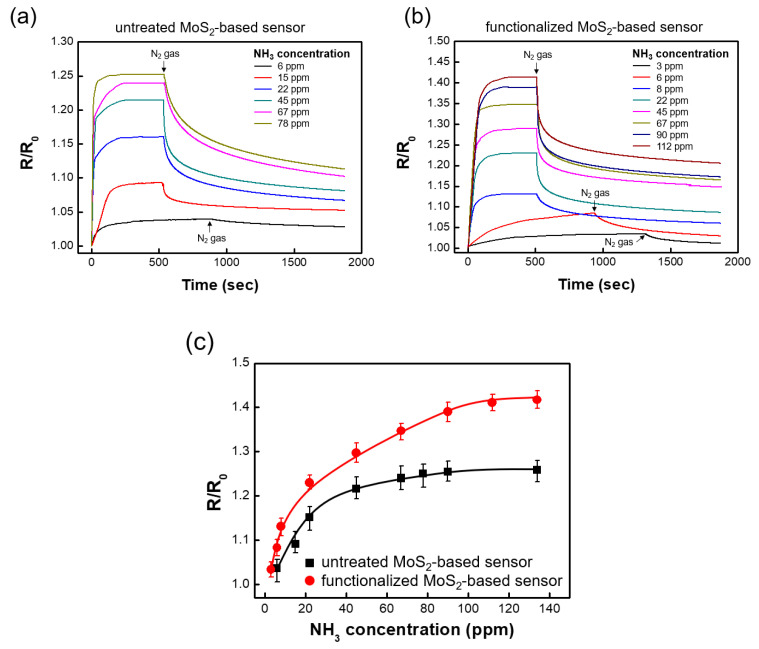
Responses of (**a**) untreated and (**b**) functionalized MoS_2_-based sensors for different NH_3_ concentrations at room temperature. (**c**) Variation in R/R_0_ of the MoS_2_ films exposed to different NH_3_ concentrations, where R_0_ is the resistance of the device in the outgassed state and R is the steady-state resistance when exposed to the NH_3_ gas.

As shown in Figure 6, the analytical performance characteristics of the functionalized MoS_2_ films were confirmed using an NH_3_ gas sensor. The electrical properties of MoS_2_ are highly sensitive to charge transfer between adsorbed molecules and the 2D films. According to theoretical studies, NH_3_ interacts weakly with perfect films and induces little charge transfer [33,34]. From the theoretical studies discussed above, it can be expected that the functionalized MoS_2_-based gas sensor has a higher sensitivity than the pristine MoS_2_-based sensor. Therefore, the adsorption of NH_3_ onto defect-free films is difficult, whereas MoS_2_ films with functional groups can easily adsorb the gas. Additionally, the adsorption barrier of functionalized MoS_2_ films is further lowered by pre-dissociated oxygen atoms, which results in an increase in the charge transfer rate [35]. Both the untreated and functionalized devices exhibited an increase in resistance upon exposure to NH_3_ gas [36]. Figure 6c presents the calibrated response curves for different gas concentrations of the untreated and functionalized MoS_2_-based sensors. The device with untreated MoS_2_ had a sensitivity of 0.0075 at a gas concentration range of 0–22 ppm. On the other hand, the functionalized MoS_2_-based sensor showed a sensitivity of 0.02 for the same range, which is 2.6 times higher. This enhanced sensitivity is attributable to a reduced electron-withdrawing ability from the formation of hydrogen bonds between the functional groups and polar NH_3_ molecules [37]. Moreover, the device with the functionalized MoS_2_ film showed a detection limit two times lower than that of the untreated device.

## 4. Conclusions

In this study, a technique for the stable functionalization of MoS_2_ was investigated. Although it is difficult to produce functional groups on 2D materials, it is possible to stably form functional groups on MoS_2_ films through treatment with weak plasma and successive exposure to MPA. Sulfur vacancies were formed on the MoS_2_ film surface by a minimal plasma treatment (10 W and 2 s), and then carboxyl groups were formed through coordinate bonds at the sulfur vacancies using an MPA solution. The stable functionalization of MoS_2_ using this strategy was confirmed by the performance of an NH_3_ sensor. The functionalized MoS_2_-based device at low gas concentrations exhibited a 2.6 times greater sensitivity than the device with the untreated MoS_2_ film since the adsorption of NH_3_ onto perfect films is not only difficult but also causes weak charge transfer. Furthermore, the MoS_2_ films with functional groups provided lower detection limits since they allow for easier gas adsorption. The proposed strategy is suitable for the fabrication of sensors based on other 2D materials in addition to MoS_2_-based devices.
